# School trajectory disruption among adolescents living with perinatal HIV receiving antiretroviral treatments: a case-control study in Thailand

**DOI:** 10.1186/s12889-021-10189-x

**Published:** 2021-01-21

**Authors:** Ophélie Merville, Patcharee Puangmala, Pranee Suksawas, Woranut Kliangpiboon, Waraporn Keawvilai, Chorkanikar Tunkam, Suvimon Yama, Usa Sukhaphan, Somporn Sathan, Siriporn Marasri, Louise Rolland-guillard, Wasna Sirirungsi, Sophie Le Cœur

**Affiliations:** 1 Institut national d’études démographiques (INED), Paris, France; 2 Phayao Provincial Hospital, Phayao, Thailand; 3 Prapokklao Hospital, Chantaburi, Thailand; 4 Nong Khai Hospital, Nong Khai, Thailand; 5 Rayong Hospital, Rayong, Thailand; 6 Sanpatong Hospital, Chiang Mai, Thailand; 7 Mae Chan Hospital, Chiang Rai, Thailand; 8 Hat Yai Hospital, Songkhla, Thailand; 9 Chiang Kham Hospital, Phayao, Thailand; 10 Bhumibol Adulyadej Hospital, Bangkok, Thailand; 11 Department of Medical Technology, Faculty of Associated Medical Science, Chiang Mai University, Chiang Mai, Thailand; 12 Institut de recherche pour le développement (IRD) UMI 174-PHPT, Chiang Mai, Thailand

**Keywords:** HIV, Thailand, Education, Adolescents, Perinatal, Case-control study

## Abstract

**Background:**

Adolescents living with perinatal HIV often experience difficult living circumstances that can impact educational achievement and thus their transition to adult life. We explored their school trajectories and evaluated the contribution of perinatal HIV-infection, in Thailand, where education is free and compulsory until the age of 15.

**Methods:**

We used data from the Teens Living with Antiretrovirals (TEEWA) study, a cross-sectional case-control study conducted from 2011 to 2014 in Thailand. Participants were 707 adolescents living with perinatal HIV (ALPHIV, cases) aged 12–19 receiving antiretroviral therapy in 19 hospitals throughout Thailand and 689 HIV-uninfected adolescents (controls) living in the same institutions or, for those living in family settings, randomly selected from the general population and individually matched for sex, age, and place of residence. School trajectory disruption was defined as ≥1 year of academic delay or as early school dropout (before 15 years of age). Logistic regression models were used to assess factors independently associated with disrupted school trajectory and to estimate the proportion of school disruption attributable to HIV-infection. We used multivariate imputations by chained equations (MICE) to manage missing data and performed two sensitivity analyses to evaluate the main model’s reliability.

**Results:**

The study population’s median age was 14.5 years (58% female). School trajectory disruption was experienced by 37% of ALPHIV and 12% of the controls. After adjusting for sociodemographic factors, ALPHIV were 5 times more likely to experience disruption than controls (***OR***_***A***_ =5.2 [3.7–7.2]). About 50% of school trajectory disruption was attributable to HIV-infection. Males and adolescents living in institutions were more likely to experience school trajectory disruption (***OR***_***A***_ =1.8 [1.3–2.4] and ***OR***_***A***_ =11.0 [7.7–15.8], respectively). Among ALPHIV, neurocognitive difficulties and growth delay were significantly associated with disruption (***OR***_***A***_ =3.3 [2.1–5.2] and ***OR***_***A***_ =1.8 [1.3–2.6], respectively). For those living in families, disruption was also associated with having a caregiver who had less than a secondary-level education (***OR***_***A***_ =2.1 [1.1–3.9]) or having experienced stigmatization (***OR***_***A***_ =1.9 [1.2–3.1]).

**Conclusions:**

HIV and contextual factors combine to aggravate the educational disadvantage among ALPHIV. The impact of this disadvantage on their life prospects, especially regarding access to higher education and professional achievement, should be further explored.

**Supplementary Information:**

The online version contains supplementary material available at 10.1186/s12889-021-10189-x.

## Introduction

With dramatic progress in access to effective antiretroviral therapies (ART), most perinatally HIV-infected children receiving treatment now reach adolescence and adulthood [1]. Despite substantial improvements in their neurodevelopment thanks to early antiretroviral therapies, impairment can persist and affect children’s academic performances [2–5].

Since the 1990s, the Royal Thai Government has emphasized the importance of education, as indicated by the launch of several major education reforms [6]. Compulsory education is free, starting between ages 6 and 7 years and lasting until the age of 15. In addition to the traditional school system, a non-formal education programme also offers basic education to youth who have dropped out the normal curriculum prematurely. However, inequalities in access to education persist, and the poorest children, those living in rural areas or those from ethnic minorities, are still disadvantaged, particularly in accessing higher education [6].

Thailand was heavily impacted by the AIDS epidemic at the end of the 1980s. Children born in the early 1990s were at high risk of mother-to-child transmission of HIV until the Thai National Mother-to-Child Transmission Prevention programme was successful in significantly reducing the risk of paediatric HIV [7]. From 2003, access to effective ART had a dramatic effect on the survival of perinatally HIV-infected children, enabling them to enter into adolescence and adulthood. At the time of this study, the first and second-line treatments were non-nucleoside reverse transcriptase inhibitor (NNRTI)-based regimen and protease inhibitor (PI)-based regimen, respectively.

Several studies have reported that despite effective therapy, perinatally HIV-infected children and adolescents often experience difficult living circumstances, such as parental loss, foster care, poverty, and stigmatization, which can have a negative impact on their educational achievements [8]. However, these studies focus on different age groups, populations, access to ART, outcomes, and comparison groups [9–19].

We hypothesize that, among adolescents living with perinatal HIV (ALPHIV), the pathways that may lead to a disrupted school trajectory include contextual factors such as their individual characteristics, socio-economic background, and school environment, income generating paid or unpaid work activities as well as specific factors related to HIV such as stigmatisation at school and health problems leading to absenteeism and academic failure (Conceptual Framework in Additional file 1).

In this analysis of the Teens Living with Antiretrovirals (TEEWA) study, school trajectories of ALPHIV in Thailand are described and compared to those of HIV-uninfected controls of the same age and living conditions, in an attempt to disentangle the contribution of HIV infection per se from that of concomitant contextual factors.

## Methods

### TEEWA study

The TEEWA study was a cross-sectional case-control study conducted in Thailand from 2011 to 2014 that aimed to investigate the overall living conditions and needs of ALPHIV.

The study included all ALPHIV – defined as the “cases” – aged 12–19 and receiving ART in one of the 19 participating hospitals throughout Thailand (11 in Northern Thailand, which is the region most affected by HIV, three in the north-east, four in the central part of the country, including Bangkok, and one in the south).

For each ALPHIV living in a family setting, one control, presumed uninfected, was randomly selected from the general population and individually matched for sex, age, and district of residence. The matching procedure was the following: for every hospital, we selected the village located in the same district where most of the surveyed ALPHIV lived with their families. In the health centre of this village, we extracted, from the computerized file of the whole population for the village, the list of adolescents of the same age and sex as the ALPHIV interviewed and then randomly selected the controls, one control per case.

ALPHIV living in institutions followed-up in the same participating hospitals were also included. They were living in eight institutions (four in the north, two in the north-east, one in the central part of the country, and one in the south). Six of these institutions were hosting both ALPHIV and known HIV-uninfected adolescents (both HIV-exposed uninfected (HEU) or non-HIV exposed children). Since a matching procedure (by institution, sex, and age) was not feasible due to the small number of children in each institution, the control group was composed of all the uninfected adolescents living in the same institution.

### Questionnaires

Each adolescent completed a self-administrated questionnaire, providing information on his/her everyday life. To prevent unintended HIV disclosure, no reference to HIV or AIDS was made in these questionnaires. The adolescents’ life history was reconstructed from structured face-to-face interviews with their primary caregivers or institution staff. Also, a medical form was completed by the hospital staff based on the ALPHIV medical records. For the control group, the adolescents’ self-administrated questionnaire was similar to that of the cases as well as the questionnaire for the caregivers except for questions related to HIV, which were omitted. The questionnaires have been already published elsewhere [20].

### Definition of school trajectory disruption

The main outcome was a school trajectory disruption defined as a composite outcome: 1) a delay of 1 or more years compared to the age expected for the school grade; or 2) a dropout before the end of compulsory education at Grade 9; or 3) an enrolment in a non-formal education programme.

To generate this composite outcome, we used the type of education (formal vs. non-formal, based on information obtained from the caregivers) and two binary variables obtained from the adolescent questionnaire: academic delay (≥ 1 year) derived from age and current grade for adolescents attending school at the time of the survey, or early school dropout derived from age and grade at the time of school termination for the others. Thus, a binary variable allows us to distinguish disrupted school trajectory from normal school progression.

### Variables

Variables obtained from the adolescent questionnaire were: sex, age (used as continuous variables), as well as variables related to their school life, such as current school attendance (yes, no), attendance in extra-curricular programmes (yes, no), friends at school (none or few vs. many), school life enjoyment (yes, no), self-reported academic performance (very poor, poor, fair, good, or excellent), history of hospitalizations (yes, no), absenteeism for medical reasons (rarely, sometimes, regularly, or for a long time), and higher education aspirations (yes, no, don’t know).

Other information was obtained from the caregiver questionnaire, such as ethnic origin (ethnic minority vs. Thai), orphan status (one or both parents deceased, both parents alive), type of caregiver (parent, grandparent, more distant relative or guardian), caregiver’s level of education (secondary school and above, primary school, never attended school), perception of the household’s financial situation (fair, good, or very good vs. difficult or very difficult), type of living area (rural, urban), type of school (public or not), any history of school grade repetition (yes, no), perception of neurocognitive difficulties experienced by the adolescents (yes, no), and knowledge of stigmatization experienced by the adolescents at school (no, yes, don’t know). However, information obtained from the institution’s staff was less detailed about the adolescents’ life history.

Finally, for ALPHIV, additional variables were obtained from the medical file: height (converted in height-for-age *z*-score (HAZ) using WHO child growth standards with stunting defined by HAZ < − 2 SD), age at HIV diagnosis (< 7.5 years, ≥ 7.5 years), age at ART initiation (< 9 years, ≥ 9 years), ART type (NNRTI-based ART vs. PI-based ART or other), most recent CD4 count (< 20%, ≥ 20%), and HIV viral load (< 1000 cp/mL, ≥ 1000 cp/mL).

### Data analysis

To compare cases and their matched controls living in family settings, McNemar’s paired test for categorical variables was used. To compare cases and controls living in institutions (not matched), the Chi-squared test was used for categorical variables. Non-parametric tests were used for continuous variables: Wilcoxon’s signed-rank test for matched samples and the Mann–Whitney–Wilcoxon test for independent samples.

For the analysis of factors associated with disrupted school trajectory, we performed bivariate and multivariable logistic regressions. To select the variables included in the multivariable logistic regressions, a conceptual framework was developed for potential pathways leading to disrupted school trajectories, based on a literature review [10–14, 16, 21–26] and on our research hypotheses (Additional file 1).

Among all adolescents surveyed, the main explanatory variable of interest was HIV-infection. Models were adjusted for the following factors: sex, age, living circumstances, and hospitalization history. We also estimated the proportion of school trajectory disruptions attributable to HIV (attributable fraction, or AF) using an approach developed by Bruzzi et al. [27]. The estimate was adjusted for potential confounders using our main logistic model.

Then the analysis focused on ALPHIV to investigate the effect of HIV-specific factors on schooling. Models were adjusted for age at ART initiation, ART type, history of hospitalization and comorbidities such as neurocognitive difficulties or growth delay defined as HAZ < − 2.

Finally, the analysis was restricted to ALPHIV living in family settings, a group for whom an additional set of contextual variables was available, such as ethnicity, type of caregiver, caregiver’s educational level, household’s financial situation, type of living area, school type, as well as stigmatization related to HIV infection.

We used multivariate imputations by chained equations to manage missing data (R “MICE” package) before fitting models. Data were analysed using R software version 3.5.3.

### Sensitivity analyses

We also performed two sensitivity analyses using different definitions of school trajectory disruption: 1) academic delay of 2 or more years or early school dropout; 2) age–grade delay as proposed by Psacharopoulos & Yang [28] using the following school-for-age (SAGE) formula:

$$ SAGE=\frac{S}{A-E}\times 100 $$

where *S* represents the number of completed school years, *A* the current age or age at school dropout, and *E* the upper age limit for primary-school admission (7 years in Thailand). For early dropout, i.e. before Grade 9, the score was calculated using the upper limit of compulsory education (age 15 in Thailand). A binary variable was created, the age-grade delay, defined as a score below 100 or attendance in a non-formal education programme.

For these two sensitivity analyses, we conducted logistic regressions adjusting for the same independent variables as in the main analysis.

Finally, we analysed the main outcome, excluding the HIV-exposed uninfected controls (HEU) to rule out the possible confounding role of parental HIV.

## Results

### Study population

**Fig. 1:**
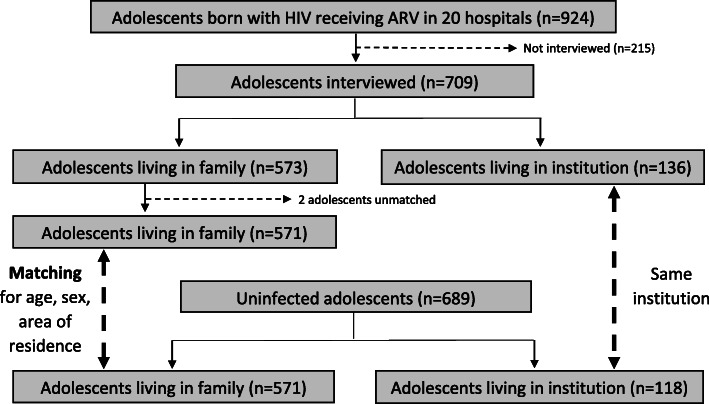
Flow chart of adolescents participating in the survey

At the time of the survey, 924 ALPHIV (12–19 years) were receiving ART in the 19 participating hospitals. Among them, 709 (77%) were interviewed with their caregiver/institutional staff, 573 living in family settings and 136 in institutions. Two adolescents living with family were excluded because they had no appropriate control in the general population. Therefore, we analysed data for 707 ALPHIV, including 571 living in family settings and 136 in institutions. The control group was composed of 689 adolescents, including 571 also living in family settings and 118 in institutions (Fig. 1).

Table 1 presents the adolescents’ sociodemographic, educational, school-life, and health characteristics. Cases and controls are compared, distinguishing those living in family settings and in institutions.

**Table 1:** Adolescents’ characteristics: Comparison of ALPHIV and controls, distinguishing adolescents living in family and in institutions

	Adolescents living in family			Adolescents living in institutions		
Variables (n in case of missing data)	ALPHIV ***N*** = 571 (%) Median (sd)	Controls ***N*** = 571 (%) Median (sd)	***p***-value	ALPHIV ***N*** = 136 (%) Median (sd)	Controls ***N*** = 118 (%) Median (sd)	***p***-value
**SOCIO-DEMOGRAPHIC CHARACTERISTICS**						
**Sex** *(male)*	238 (41.7)	238 (41.7)		68 (50.0)	59 (50.0)	
**Age**	14.4 (2.9)	14.4 (2.9)		14.8 (3.1)	14.6 (3.4)	
**Orphan** *(one or both parents)*	493 (86.3)	65 (11.4)	<0.001^†^	131 (96.3)	88 (74.6)	<0.001^§^
**Ethnic minority** (475,571,129,116)	15 (2.6)	29 (5.1)	0.12^§^	11 (8.1)	37 (31.4)	<0.001^§^
**Type of caregiver** (560, 570)						
Parent	179 (31.3)	415 (72.7)	<0.001^*¥*^	**–**	**–**	
Grandparent	213 (37.3)	89 (15.6)		**–**	**–**	
More distant relative or guardian	168 (29.4)	66 (11.6)		**–**	**–**	
**Caregiver’s level of education**						
Secondary school and above	108 (18.9)	211 (37.0)	<0.001^*¥*^	**–**	**–**	
Primary school	379 (66.4)	325 (56.9)		**–**	**–**	
Never attended school	84 (14.7)	35 (6.1)		**–**	**–**	
**Household’s financial situation difficult**	207 (36.3)	109 (19.1)	<0.001^†^	**–**	**–**	
**Living area rural** (569,569)	442 (77.4)	458 (80.2)	0.16^†^	**–**	**–**	
**EDUCATION**						
**Current school attendance**	472 (82.7)	538 (94.2)	<0.001^†^	130 (95.6)	118 (100)	0.03^‡^
**Disrupted school trajectory** (564,571,135,118)	157 (27.5)	32 (5.6)	<0.001^†^	101 (74.3)	52 (44.1)	<0.001^§^
Academic delay (≥ 1 year) (564,571,135,118)	96 (16.8)	21 (3.7)	<0.001^†^	96 (70.6)	52 (44.1)	<0.001^§^
Early school dropout	61 (10.7)	11 (1.9)	<0.001^†^	5 (3.7)	0 (0)	0.06^‡^
**SCHOOL LIFE**						
**In public school** (570,571,132,115)	532 (93.2)	523 (91.6)	0.29^†^	82 (60.3)	90 (76.3)	0.01^§^
**Attendance in extra-curricular school** (569,571,135,118)	306 (53.4)	378 (66.2)	<0.001^†^	85 (62.5)	84 (71.2)	0.17^§^
**No or few friends at school** (567,571,136,118)	64 (11.2)	23 (4.0)	<0.001^†^	17 (12.5)	31 (26.3)	0.01^§^
**Lack of school life enjoyment** (566,571,136,118)	75 (13.1)	56 (9.8)	0.06^†^	19 (14.0)	25 (21.2)	0.13^§^
**Self-reported academic performance** (564,571,136,118)						
Mediocre / poor	26 (4.6)	13 (2.3)	0.07^*¥*^	12 (8.8)	11 (9.3)	0.76^§^
Fair	282 (49.4)	279 (48.9)		59 (43.4)	56 (47.5)	
Good / excellent	258 (45.2)	279 (48.9)		65 (47.8)	51 (43.2)	
**Absenteeism for medical reasons** (560,570,135,118)						
Rarely	195 (34.2)	332 (58.1)	<0.001^*¥*^	60 (44.1)	67 (56.8)	0.13^§^
Sometimes	234 (41.0)	175 (30.6)		52 (38.2)	33 (28.0)	
Regularly / for a long time	131 (22.9)	63 (11.0)		23 (16.9)	18 (15.3)	
**Grade repetition** (567,571,136,118)	87 (15.2)	19 (3.3)	<0.001^†^	–	–	
**Higher education aspirations**						
Yes	285 (49.9)	411 (72.0)	<0.001^*¥*^	74 (54.4)	75 (63.6)	0.32^§^
No	104 (18.2)	36 (6.3)		21 (15.4)	16 (13.6)	
Don’t know	182 (31.9)	124 (21.7)		41 (30.1)	27 (22.9)	
**Stigmatization experience(s) at school** (564)	134 (23.5)	–		–	–	
**HEALTH**						
**History of hospitalizations** (570,571,136,118)	347 (60.8)	225 (39.4)	<0.001^†^	76 (55.9)	36 (30.5)	<0.001^§^
**Neurocognitive difficulties** (481,571,136,118)	87 (15.2)	5 (0.9)	<0.001^§^	25 (18.4)	8 (6.4)	0.006^§^
**HAZ < −2** (569,128)	240 (42.0)	–		46 (33.8)	–	
**Age at ART initiation** (549,121)	9.0 (3.1)	–		8.6 (3.5)	–	
**Age at diagnosis** (563,106)	7.5 (3.8)	–		8.3 (4.0)	–	
**NNRTI based ART** (571,131)	421 (73.7)	–		87 (64.0)	–	
**Viral load ≥ 1000 cp/mL** (569,130)	103 (18.0)	–		3 (2.2)	–	
**CD4 count < 20%** (568,130)	120 (21.0)	–		13 (9.6)	–	

^†^: Mc Nemar test; ^‡^: Fisher’s exact test (when conditions for the application of Chi-squared test are not met); ^§^: Chi-squared test; ^¥^: Stuart-Marxwell test (for matched analyses with variables with more than 2 categories)

### Sociodemographic profile of ALPHIV and controls

#### Adolescents living in family settings

Both groups (ALPHIV and controls) were composed of males (42%) and females (58%) with a median age of 14.4 years.

Most ALPHIV (86%) had lost one or both parents, while most controls had not (11%; *p* < 0.001). Among ALPHIV, only one-third (31%) had a parent as the primary caregiver compared to 73% of controls. Educational level of caregivers was significantly lower among ALPHIV than among controls, with 19% of ALPHIV caregivers having had attended secondary school or above versus 37% of the controls’ caregivers. The household financial situation was significantly more often perceived as “difficult” among ALPHIV than controls (36% vs. 19%, *p* < 0.001).

#### Adolescents living in institutions

Both groups were composed in equal proportions of males and females with a median age of 14.8 years. Almost all ALPHIV had lost one or both parents, whereas the proportion was approximately three-quarters for the control group (*p* < 0.001). Controls were significantly more often members of ethnic minorities than were ALPHIV (31% vs. 8%, *p* < 0.001).

### Educational and school-life characteristics of ALPHIV and controls

#### Adolescents living in family settings

At the time of the survey, ALPHIV were less likely to be attending school than their peers in the general population (83% vs. 94%, *p* < 0.001). Disrupted school trajectories were more frequent among cases than controls (27% vs. 6%, *p* < 0.001). Most (> 90%) cases and controls living in family settings were enrolled in public schools, but ALPHIV were less likely to attend extracurricular programmes (53% vs. 66%, *p* < 0.001). Cases reported having few or no friends more often than controls (11% vs. 4%, *p* < 0.001). However, school-life enjoyment and self-reported academic performance did not differ between cases and controls. Significantly more cases than controls mentioned frequent absenteeism for medical reasons (23% vs. 11%, *p* < 0.001), and they were more likely to have repeated a grade (15% vs. 3%, *p* < 0.001). ALPHIV were more often reluctant or unsure about pursuing higher education than were controls (50% vs. 28%, *p* < 0.001). Finally, according to their caregiver, 24% of ALPHIV had experienced stigmatization at school in the form of bullying, violent or humiliating behaviours or exclusion.

#### Adolescents living in institutions

At the time of the survey, almost all adolescents (> 95%) living in institutions were currently attending school. Unlike the group of adolescents living in family settings, most school-life characteristics did not differ between cases and controls. Disrupted school trajectories were, however, more frequent among cases than controls, although the percentages were high in both groups (74% vs. 44%, *p* < 0.001). Interestingly, cases were more likely to report having many friends at school (88% vs. 74%, *p* = 0.01) and to be attending a private school (36.8% vs. 21.2%, *p* = 0.01). The control group comprised a small number (*n* = 16) of HIV-exposed uninfected (HEU) adolescents (born from HIV-infected mothers). The percentage of school trajectory disruption among HEU was slightly lower than among the HIV non-exposed uninfected controls (31% vs. 45%, data not shown).

### Health situation of ALPHIV and controls

#### Adolescents living in family settings

Having a hospitalization history and neurocognitive difficulties were more frequent among ALPHIV than controls (61% vs. 39%, *p* < 0.001 and 15% vs. 1%, *p* < 0.001, respectively). Moreover, 42% of ALPHIV suffered from growth delay (HAZ < 2). Median ages at HIV diagnosis and ART initiation were 7.5 and 9.0 years, respectively.

At the time of the survey, 74% of ALPHIV were receiving NNRTI-based ART and 18% of ALPHIV had a viral load ≥1000cp/mL and 21% a CD4 count < 20%.

#### Adolescents living in institutions

Here also, a history of hospitalizations and neurocognitive difficulties were more frequent among ALPHIV than controls according to institutional staff (56% vs. 31%, *p* < 0.001 and 18% vs. 6%, *p* < 0.001, respectively). Thirty-four percent of ALPHIV had an HAZ of < − 2. The median age at HIV diagnosis was 8.3 and 8.6 years at ART initiation.

At the time of the survey, 69% of ALPHIV were receiving NNRTI-based ART. Only 2% of ALPHIV had a viral load ≥1000cp/mL and 9% had a CD4 count < 20%.

### Factors associated with school trajectory disruption

#### Among all adolescents surveyed: cases or controls

The multivariable analysis indicated that the risk of having a disrupted school trajectory was 5 times higher among ALPHIV (*OR*_*A*_ = 5.15 [3.72–7.23], *p* < 0.001) compared to controls (Table 2). Other factors associated with school trajectory disruption were: male sex (*OR*_*A*_ = 1.76 [1.32–2.35], *p* < 0.001), living in an institution (*OR*_*A*_ = 10.98 [7.72–15.80], *p* < 0.001), or being cared for by a distant relative or guardian (*OR*_*A*_ = 1.49 [1.03–2.15], *p* = 0.03), compared to being cared by parents and grand-parents, and history of hospitalizations (*OR*_*A*_ = 1.46 [1.09–1.97], *p* = 0.01). Age was also significantly associated with a disrupted school trajectory, with a 22% increase per year (*OR*_*A*_ = 1.22 [1.13–1.31], *p* < 0.001).

**Table 2:** Factors associated with school trajectory disruption among all adolescents (cases and controls): bivariate/multivariable analyses

	Disrupted school trajectory		Bivariate analysis	Multivariable analysis	
	No ***N*** = 1046 N (%) / Median (sd)	Yes ***N*** = 342 N (%) / Median (sd)	ORB (IC95%)	ORA (IC95%)	***p***-value†
HIV status					
Controls	605 (87.8)	84 (12.2)	1	1	
ALPHIV	441 (63.1)	258 (36.9)	4.21 [3.20–5.55]	5.15 [3.72–7.23]	<0.001
Sex					
Female	628 (79.6)	161 (20.4)	1	1	
Male	418 (69.8)	181 (30.2)	1.69 [1.32–2.16]	1.76 [1.32–2.35]	< 0.001
Type of caregiver					
Parent or grandparent	765 (85.9)	126 (14.1)	1	1	
More distant relative or guardian	172 (74.1)	60 (25.9)	2.12 [1.49–3.00]	1.49 [1.03–2.15]	0.03
Institution staff	100 (39.5)	153 (60.5)	9.29 [6.78–12.72]	10.98 [7.72–15.80]	<0.001
History of hospitalizations					
No	567 (79.9)	143 (20.1)	1	1	
Yes	479 (70.8)	198 (29.2)	1.64 [1.28–2.10]	1.46 [1.09–1.97]	0.01
Age (years)	14.6 (3.4)	15.2 (3.8)	1.19 [1.11–1.26]	1.22 [1.13–1.31]	<0.001

^†^: Wald test

We estimated that about half of school trajectory disruptions could be attributed to HIV-infection (*AF* = 0.47 [0.38–0.55], *p* < 0.001).

#### Among all ALPHIV living in family settings or in institutions

Sociodemographic factors such as male sex (*OR*_*A*_ = 1.57 [1.09–2.27], *p* = 0.02), age (*OR*_*A*_ = 1.21 [1.08–1.35], *p* < 0.001), and living in an institution (*OR*_*A*_ = 9.96 [6.20–16.37], *p* < 0.001) were significantly associated with disrupted school trajectories (Table 3). Adolescents who mentioned a history of hospitalizations were more likely to experience school trajectory disruption (*OR*_*A*_ = 1.52 [1.05–2.22], *p* = 0.03), as were those with neurocognitive difficulties (*OR*_*A*_ = 3.29 [2.11–5.19], *p* < 0.001) and those with a HAZ < -2 (*OR*_*A*_ = 1.81 [1.25–2.62], *p* = 0.001). Age at ART initiation, type of ART (first- or second-line), and type of caregiver were not associated with disrupted school trajectories, after adjustment for the other factors.

**Table 3:** Factors associated with school trajectory disruption among ALPHIV living in family and institutions: bivariate/multivariable analyses

	Disrupted school trajectory		Bivariate analysis	Multivariable analysis	
	No ***N*** = 441 N (%) / Median (sd)	Yes ***N*** = 258 N (%) / Median (sd)	ORB (IC95%)	ORA (IC95%)	***p***-value†
Sex					
Female	273 (68.8)	124 (31.2)	1	1	
Male	168 (55.6)	134 (44.4)	1.76 [1.29–2.40]	1.57 [1.09–2.27]	0.02
Age (years)	14.4 (3.5)	15.1 (3.5)	1.20 [1.10–1.30]	1.21 [1.08–1.35]	<0.001
Type of caregiver					
Parent or grandparent	287 (74.2)	100 (25.8)	1	1	
More distant relative or guardian	112 (67.5)	54 (32.5)	1.38 [0.93–2.06]	1.30 [0.85–1.98]	0.22
Institution staff	34 (25.2)	101 (74.8)	8.52 [5.43–13.4]	9.96 [6.20–16.37]	<0.001
History of hospitalizations					
No	192 (68.1)	90 (31.9)	1	1	
Yes	249 (59.9)	167 (40.1)	1.43 [1.04–1.96]	1.52 [1.05–2.22]	0.03
Neurocognitive difficulties					
No	325 (65.0)	175 (35.0)	1	1	
Yes	45 (40.9)	65 (59.1)	2.76 [1.76–4.09]	3.29 [2.11–5.19]	<0.001
HAZ					
≥ −2	277 (68.4)	128 (31.6)	1	1	
< − 2	162 (57.0)	122 (43.0)	1.63 [1.19–2.23]	1.81 [1.25–2.62]	0.001
Age at ART initiation					
< 9 years-old	226 (69.3)	100 (30.7)	1	1	
≥ 9 years-old	201 (59.8)	135 (40.2)	1.52 [1.10–2.09]	1.43 [0.93–2.19]	0.10
ART type					
NNRTI based	333 (66.1)	171 (33.9)	1	1	
PI based (or other)	108 (57.4)	80 (42.6)	1.44 [1.02–2.03]	1.42 [0.95–2.12]	0.09

^†^: Wald test

#### Among ALPHIV living in family settings

In this subpopulation, age and male sex were also significantly associated with disrupted school trajectories, as well as having neurocognitive difficulties and growth delay (Table 4). Additional contextual factors, such as being from an ethnic minority (*OR*_*A*_ = 3.14 [1.00–9.75], *p* = 0.05), having a caregiver with less than secondary-level education (*OR*_*A*_ = 2.05 [1.12–3.88], *p* = 0.02 for primary school dropout and *OR*_*A*_ = 2.37 [1.05–5.41], *p* = 0.04 for those who had never attended school), and experience of stigmatization at school (*OR*_*A*_ = 1.94 [1.21–3.10], *p* = 0.01) were significantly associated with a disrupted school trajectory. Adolescents on second-line treatment were more likely to experience school trajectory disruption (*OR*_*A*_ = 1.66 [1.04–2.64], *p* = 0.03), but there was no significant difference according to age at ART initiation. There was no significant association with the type of caregiver, household’s financial situation, type of school, type of living area, and history of hospitalization.

**Table 4:** Factors associated with school trajectory disruption among ALPHIV living in family: bivariate/multivariable analyses

	Disrupted school trajectory		Bivariate analysis	Multivariable analysis	
	No ***N*** = 407 N (%) / Median (sd)	Yes ***N*** = 157 N (%) / Median (sd)	ORB (IC95%)	ORA (IC95%)	***p***-value†
Sex					
Female	256 (77.8)	73 (22.2)	1	1	
Male	151 (64.3)	84 (35.7)	1.79 [1.17–2.73]	1.81 [1.18–2.79]	0.01
Age (years)	14.4 (3.4)	15.2 (3.5)	1.23 [1.12–1.36]	1.27 [1.12–1.45]	<0.001
Neurocognitive difficulties					
No	295 (75.6)	95 (24.4)	1	1	
Yes	41 (48.2)	44 (51.8)	3.33 [2.05–5.41]	3.34 [2.00–5.61]	<0.001
HAZ					
≥ −2	254 (78.4)	70 (21.6)	1	1	
< −2	151 (63.4)	87 (36.6)	2.09 [1.44–3.04]	1.80 [1.18–2.76]	0.01
Ethnic origin					
Thai	331 (72.7)	124 (27.3)	1	1	
Ethnic minority	7 (46.7)	8 (53.3)	3.05 [1.08–8.59]	3.14 [1.00–9.75]	0.05
Caregiver’s level of education					
Secondary school and above	89 (82.4)	19 (17.6)	1	1	
Primary school	261 (70.0)	112 (30.0)	2.01 [1.17–3.46]	2.05 [1.12–3.88]	0.02
Never attended school	57 (68.7)	26 (31.3)	2.14 [1.08–4.21]	2.37 [1.05–5.41]	0.04
Stigmatization experience(s) at school					
No or don’t know	326 (75.8)	104 (24.2)	1	1	
Yes once or more	81 (60.4)	53 (39.6)	2.05 [1.36;3.09]	1.94 [1.21–3.10]	0.01
ART type					
NNRTI based	310 (74.2)	108 (25.8)	1	1	
PI based (or other)	97 (66.4)	49 (33.6)	1.45 [0.96–2.18]	1.66 [1.04–2.64]	0.03
Age at ART initiation					
< 9 years	208 (78.5)	57 (21.5)	1	1	
≥ 9 years	186 (67.1)	91 (32.9)	1.79 [1.21;2.63]	1.49 [0.90–2.46]	0.12
Type of caregiver					
Parent	140 (78.2)	39 (21.8)	1	1	
Grandparents	147 (70.7)	61 (29.3)	1.49 [0.94–2.37]	1.03 [0.60–1.77]	0.93
More distant relative or guardian	112 (67.5)	54 (32.5)	1.73 [1.07–2.80]	1.53 [0.89–2.63]	0.12
Household’s financial situation					
Fair / good / very good	259 (71.9)	101 (28.1)	1	1	
Difficult / very difficult	148 (72.5)	56 (27.5)	0.97 [0.66–1.42]	0.92 [0.59–1.42]	0.70
Public school					
Yes	383 (72.5)	145 (27.5)	1	1	
No	24 (66.7)	12 (33.3)	1.32 [0.64–2.71]	0.99 [0.42–2.20]	0.98
Type of living area					
Rural	317 (72.4)	121 (27.6)	1	1	
Urban	88 (71.0)	36 (29.0)	1.07 [0.69–1.67]	1.49 [0.90–2.45]	0.12
History of hospitalizations					
No	173 (77.9)	49 (22.1)	1	1	
Yes	234 (68.6)	107 (31.4)	1.61 [1.09–2.39]	1.40 [0.91–2.17]	0.13

^†^: Wald test

In the sensitivity analyses, using 2 or more years of academic delay as a threshold or the age–grade delay, results were similar to those obtained for our main outcome (Additional files 2, 3, 4). Also, after excluding the HEU from the control group, results remained unchanged (Additional file 5).

## Discussion

Our results indicate that, after adjustment for sociodemographic factors, ALPHIV were 5 times more likely to experience school trajectory disruption than controls, and half of school trajectory disruptions were attributable to HIV-infection. Furthermore, all other factors being equal, for ALPHIV living in institutions, the risk of a disrupted school trajectory was 10 times greater than that of controls. Among ALPHIV, school trajectory disruption was also significantly associated with HIV comorbidities, such as neurocognitive difficulties or growth delay, and stigmatization experiences.

In the TEEWA survey, school attendance was high, above 85% for both ALPHIV and controls, in the upper range compared to other studies’ results [10–14, 16]. However, among adolescents living in family settings, school attendance was lower among ALPHIV than among controls from the general population (83% vs. 94%, respectively). Also, the proportion of school trajectory disruption among ALPHIV was significantly higher than among controls (17% vs. 4%, respectively) and similar to the proportion of 20% found in another study among HIV-infected children aged 6–12 in Thailand [29].

For adolescents living in institutions, school attendance was almost universal because of the strict institutional adherence to administrative rules. However, the risk of school trajectory disruption was much higher than for those living in family settings. Children living in institutions often experience chaotic life trajectories that include parental loss, caregiver turnover, neglect by relatives, or poverty, which makes them particularly vulnerable.

As usually observed, males were more prone to disrupted school trajectory than females [10, 15, 16, 21–23]. Furthermore, ALPHIV from ethnic minorities were more likely to experience disrupted school trajectories than their peers, probably because of Thai-language difficulties or administrative issues causing delays in school enrolment [30].

Some studies have highlighted the negative effect of adverse living conditions on school enrolment and academic performance [8, 10–12, 19, 21–24, 26, 29]. In our study, having a caregiver who had less than a secondary-level education was significantly associated with disrupted school trajectories, but no significant difference was found according to the household’s financial situation or type of living area (rural or urban). This result could be a consequence of the recent policies to improve access to education in Thailand, with free, compulsory primary and lower-secondary education [6, 31].

Several studies carried out in North America have focused on academic achievement among HIV-infected or affected children [15, 18, 19]. Garvie et al. have shown that academic performance of HIV-infected and HIV-exposed uninfected (HEU) children aged 7–16 years was significantly lower than the general population standards but not significantly different from each other [19]. In contrast, in our study, the proportion of school trajectory disruptions among adolescents living in institutions was slightly lower among HEU than HIV-uninfected controls, suggesting the main role of HIV-infection per se to educational disadvantage.

Studies carried out in low-income countries also found a significant association between HIV-infection and disrupted schooling after adjustment for socio-economic factors [10, 11]. In a recent study in South Africa, Fotso et al. found that HIV-infection significantly reduced adolescent educational attainment and that contextual factors could only explain 18% of the educational attainment gap [10]. While neurocognitive impairments related to HIV-infection can directly affect academic achievement, school absenteeism because of frequent illness or HIV-related stigmatization may mediate the negative effect of HIV-infection on school life [13, 14]. In the TEEWA study, about 20% of ALPHIV mentioned episodes or extended periods of school absenteeism for medical reasons. In TEEWA, after adjustment, neurocognitive difficulties and growth delay were significantly associated with disrupted school trajectories. This finding confirms the results from a recent cross-sectional study, according to which HIV-infected children were not only more likely to repeat a grade but had an elevated risk of social and educational exclusion if they were disabled [14].

In a literature review, Smith et al. emphasized the importance of early effective ART to reduce the negative effect of HIV-infection on neurodevelopment [2]. In the TEEWA study, after adjustment, delayed ART initiation was not associated with school trajectory disruption. However, the median age at ART initiation was relatively late for all children, at 9 years.

Finally, as in other studies [13, 14], we found that stigmatization was significantly associated with school trajectory disruption. These traumatic events, instigated by teachers, other students, or their parents, can lead to academic failure or school dropout. Yet, experience of stigmatization was probably under-reported by caregivers who may not have been informed by the children of all stigmatization episodes.

Our study presents some limitations. First, participating hospitals were not randomly selected based on regional HIV prevalence, limiting our ability to generalize the findings to the country level. Nevertheless, the survey coverage, around 10% of ALPHIV aged 12–19 and receiving ART in Thailand at the time, as well as the geographical distribution of the hospitals across the country is sufficiently representative of this population. Second, considering that most perinatally HIV-infected children in this generation had no access to ART in early childhood, our survey comprised a selected population of survivors. Third, we could not ascertain the HIV status of the controls in the general population and assumed they were HIV negative. At the time of the survey, HIV prevalence in the Thai general population was below 2% [7]; therefore the probability of randomly selecting HIV-infected adolescents was low. Indeed, during the survey, two adolescents who self-reported HIV infection, were excluded from the control group. Fourth, for the institutional settings, matching cases and controls was not feasible and the procedure to create a reference group was different from that used among adolescents living in family settings, and we cannot rule out a possible clustering effect.

Our study targeted all the ALPHIV followed in the study’s hospitals, whatever their family or institutional living circumstances. Yet, subgroup analyses on living circumstances were performed because some variables were not obtainable from the institution’s staff, possibly reducing the statistical power of the analyses. According to the size of ALPHIV population, we were limited in the number of variables to include in our model and were willing to avoid any collinearity. Therefore, we decided to use the history of hospitalization as a marker of the past health history, a variable which was also available for the controls.

Our survey covered a wide age range of adolescents facing different challenges. Younger participants were enrolled in primary school, while older participants had reached nearly the end of compulsory education. Our relatively large sample, however, and the results of our sensitivity analyses strengthen our findings.

Finally, we created a composite outcome, including an academic delay of ≥1 year and early school dropout, to investigate factors associated with a disrupted school trajectory. We assumed these events derived from similar mechanisms of academic failure, which is supported by the literature [26]. However, academic delay may result from other processes, such as delayed school entry, school interruption, or grade repetition, all of which may be compounded.

## Conclusions

Our findings highlight the educational disadvantage experienced by ALPHIV because of medical issues related to HIV, coupled with contextual factors. But as they are entering adulthood, their situation should be re-examined and their integration into society assessed [32].

ALPHIV have often lived through traumatic events, such as parental loss, caregiver turn-over, institutional placement, stigmatization, and experienced health problems interfering with their schooling. Specific educational support could be offered by social, educational, and health services to prevent academic failure and early school dropout. HIV/AIDS public awareness programmes, particularly in schools, would combat the discrimination experienced by ALPHIV and promote their well-being.

Today, thanks to the outstanding success of the HIV mother-to-child transmission prevention programmes in Thailand, few children born from HIV-infected mothers are HIV-infected themselves [7]. Our study was carried out when ART provision was still limited, and most adolescents had initiated their treatment late in childhood. It would be worth reconsidering whether this educational disadvantage persists in the new generation of ALPHIV who will have received ART earlier in life.

## Supplementary Information

**Additional file 1.** Conceptual framework for pathways potentially leading to a disrupted school trajectory (supplementary figure).

**Additional file 2.** Factors associated with school trajectory disruption among all adolescents surveyed: sensitivity analysis using ≥2-years academic delay as threshold, and the age-grade delay.

**Additional file 3.** Factors associated with school trajectory disruption among ALPHIV living in family and institutions: sensitivity analysis using ≥2-years academic delay as threshold, and the age-grade delay.

**Additional file 4.** Factors associated with school trajectory disruption among ALPHIV living in family: sensitivity analysis using ≥2-years academic delay as threshold, and the age-grade delay.

**Additional file 5.** Factors associated with school trajectory disruption among all adolescents excluding HEU: multivariable analysis.

## Data Availability

The datasets used and/or analysed here are available from the corresponding author upon reasonable request.

## Abbreviations

AIDS: Acquired immune deficiency syndrome
ALPHIV: Adolescents living with perinatal HIV
ART: Antiretroviral therapy
HAZ: Height-for-age *z*-score
HEU: HIV-exposed uninfected controls
HIV: Human immunodeficiency virus
NNRTI: Non-nucleoside reverse transcriptase inhibitor
PI: Protease inhibitor
