# Supplementary figures and images for: School trajectory disruption among adolescents living with perinatal HIV receiving antiretroviral treatments: a case-control study in Thailand
Source: BMC Public Health. 2021 Jan 21;21:189. doi: 10.1186/s12889-021-10189-x (PMC7818931; doi:10.1186/s12889-021-10189-x)

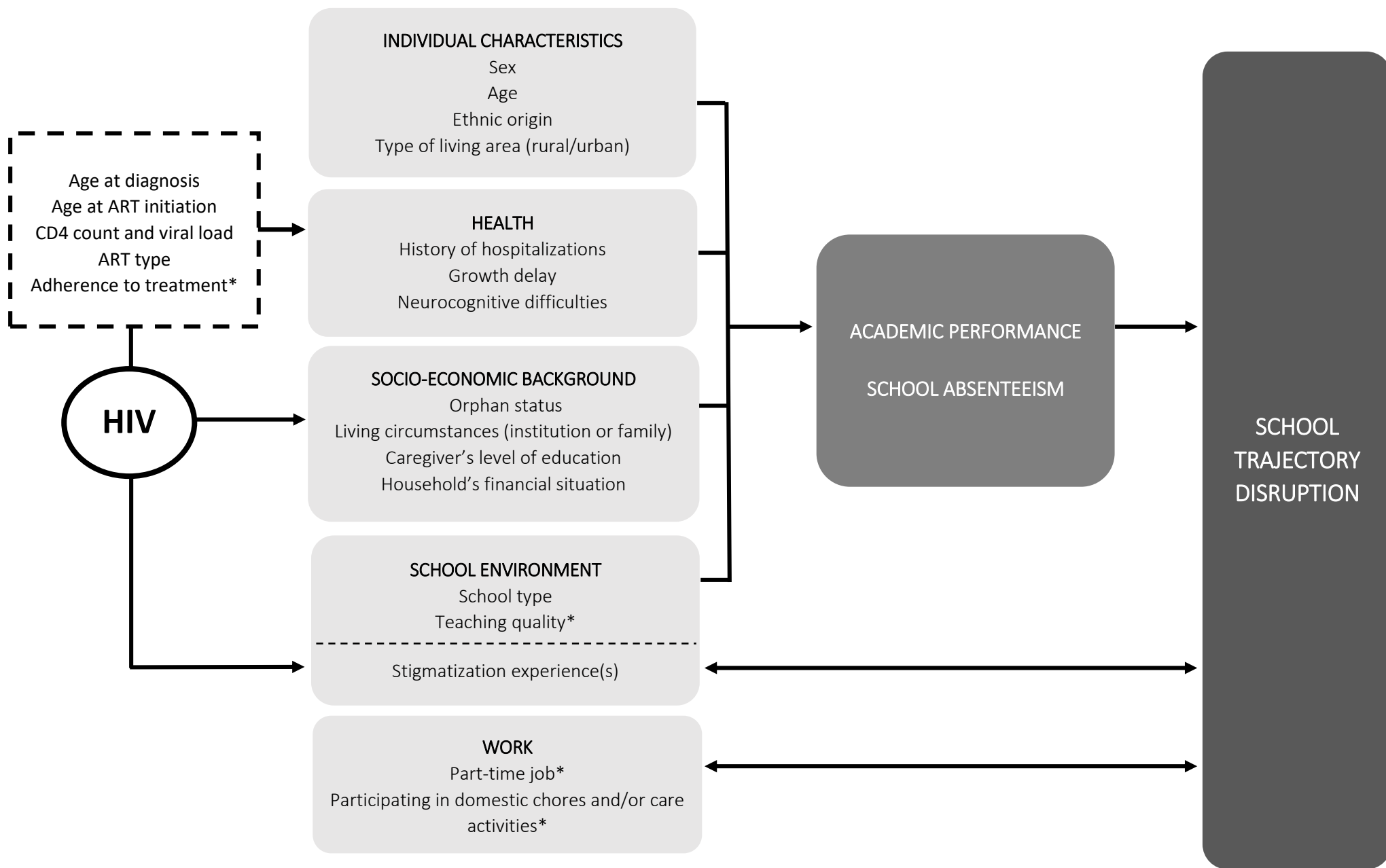

Supplement: Supplementary file 1 — Additional file 1. Conceptual framework for pathways potentially leading to a disrupted school trajectory (supplementary figure). [file 12889_2021_10189_MOESM1_ESM.pdf]
