# Supplementary material for: School trajectory disruption among adolescents living with perinatal HIV receiving antiretroviral treatments: a case-control study in Thailand
Source: BMC Public Health. 2021 Jan 21;21:189. doi: 10.1186/s12889-021-10189-x (PMC7818931; doi:10.1186/s12889-021-10189-x)
Supplement: Supplementary file 2 — Additional file 2. Factors associated with school trajectory disruption among all adolescents surveyed: sensitivity analysis using ≥2-years academic delay as threshold, and the age-grade delay. [file 12889_2021_10189_MOESM2_ESM.docx]

|  |  | **All adolescents surveyed (n=1388)** | | | | |
| --- | --- | --- | --- | --- | --- | --- |
|  |  | **Academic delay (≥ 2 years) or dropout** | |  | **Age-grade delay** | |
|  |  | **ORA (IC95%)** | **p-value**† |  | **ORA (IC95%)** | **p-value**† |
| HIV status |  |  |  |  |  |  |
| Controls |  | 1 |  |  | 1 |  |
| ALPHIV |  | 5.65 [3.85-8.46] | <0.001 |  | 5.05 [3.60-7.19] | <0.001 |
|  |  |  |  |  |  |  |
| Sex |  |  |  |  |  |  |
| Female |  | 1 |  |  | 1 |  |
| Male |  | 1.72 [1.25-2.38] | <0.001 |  | 1.70 [1.26-2.29] | <0.001 |
|  |  |  |  |  |  |  |
| Type of caregiver | |  |  |  |  |  |
| Parent or grandparent | | 1 |  |  | 1 |  |
| More distant relative or guardian | | 1.32 [0.86-2.00] | 0.20 |  | 1.50 [1.02-2.20] | 0.04 |
| Institution staff |  | 4.68 [3.23-6.80] | <0.001 |  | 12.6 [8.83-18.27] | <0.001 |
|  |  |  |  |  |  |  |
| History of hospitalizations | |  | | | | |
| No |  | 1 |  |  | 1 |  |
| Yes |  | 1.43 [1.03-2.00] | 0.03 |  | 1.34 [0.99-1.82] | 0.06 |
|  |  |  |  |  |  |  |
| Age (years) |  | 1.20 [1.11-1.31] | <0.001 |  | 1.22 [1.13-1.32] | <0.001 |

**Additional file 2. Factors associated with school trajectory disruption among all adolescents surveyed: sensitivity analysis using ≥ 2-years academic delay as threshold, and the age-grade delay**

${}^{\dagger}$: Wald test
