# Supplementary material for: School trajectory disruption among adolescents living with perinatal HIV receiving antiretroviral treatments: a case-control study in Thailand
Source: BMC Public Health. 2021 Jan 21;21:189. doi: 10.1186/s12889-021-10189-x (PMC7818931; doi:10.1186/s12889-021-10189-x)
Supplement: Supplementary file 3 — Additional file 3. Factors associated with school trajectory disruption among ALPHIV living in family and institutions: sensitivity analysis using ≥2-years academic delay as threshold, and the age-grade delay. [file 12889_2021_10189_MOESM3_ESM.docx]

|  |  | **ALPHIV living in family settings and institutions (n=699)** | | | | |
| --- | --- | --- | --- | --- | --- | --- |
|  |  | **Academic delay (≥ 2 years) or dropout** | |  | **Age-grade delay** | |
|  |  | **ORA (IC95%)** | **p-value**† |  | **ORA (IC95%)** | **p-value**† |
| Sex |  |  |  |  |  |  |
| Female |  | 1 |  |  | 1 |  |
| Male |  | 1.32 [0.90-1.94] | 0.16 |  | 1.62 [1.12-2.37] | 0.01 |
|  |  |  |  |  |  |  |
| Age (years) |  | 1.23 [1.10-1.38] | <0.001 |  | 1.19 [1.07-1.33] | 0.002 |
|  |  |  |  |  |  |  |
| Type of caregiver |  |  |  |  |  |  |
| Parent or grandparent | | 1 |  |  | 1 |  |
| More distant relative or guardian | | 1.12 [0.70-1.79] | 0.63 |  | 1.25 [0.80-1.92] | 0.32 |
| Institution staff |  | 4.61 [2.94-7.28] | <0.001 |  | 11.31 [7.02-18.7] | <0.001 |
|  |  |  |  |  |  |  |
| History of hospitalizations | |  |  |  |  |  |
| No |  | 1 |  |  | 1 |  |
| Yes |  | 1.30 [0.88-1.94] | 0.19 |  | 1.45 [0.99-2.13] | 0.06 |
|  |  |  |  |  |  |  |
| Neurocognitive difficulties |  |  |  |  |  |  |
| No |  | 1 |  |  | 1 |  |
| Yes |  | 3.20 [2.03-5.04] | <0.001 |  | 2.67 [1.70-4.22] | <0.001 |
|  |  |  |  |  |  |  |
| HAZ |  |  |  |  |  |  |
| ≥ -2 |  | 1 |  |  | 1 |  |
| < -2 |  | 1.84 [1.25-2.71] | 0.002 |  | 1.85 [1.27-2.71] | 0.001 |
|  |  |  |  |  |  |  |
| Age at ART initiation |  |  |  |  |  |  |
| < 9 years-old |  | 1 |  |  | 1 |  |
| ≥ 9 years-old |  | 1.04 [0.66-1.64] | 0.86 |  | 1.56 [1.01-2.43] | 0.05 |
|  |  |  |  |  |  |  |
| ART type |  |  |  |  |  |  |
| NNRTI based |  | 1 |  |  | 1 |  |
| PI based (or other) |  | 1.49 [0.98-2.24] | 0.06 |  | 1.52 [1.01-2.28] | 0.05 |
|  |  |  |  |  |  |  |

**Additional file 3. Factors associated with school trajectory disruption among ALPHIV living in family and institutions: sensitivity analysis using ≥ 2-years academic delay as threshold, and the age-grade delay**

${}^{\dagger}$: Wald test
