# Supplementary material for: School trajectory disruption among adolescents living with perinatal HIV receiving antiretroviral treatments: a case-control study in Thailand
Source: BMC Public Health. 2021 Jan 21;21:189. doi: 10.1186/s12889-021-10189-x (PMC7818931; doi:10.1186/s12889-021-10189-x)
Supplement: Supplementary file 4 — Additional file 4. Factors associated with school trajectory disruption among ALPHIV living in family: sensitivity analysis using ≥2-years academic delay as threshold, and the age-grade delay. [file 12889_2021_10189_MOESM4_ESM.docx]

**Additional file 4. Factors associated with school trajectory disruption among ALPHIV living in family: sensitivity analysis using ≥ 2-years academic delay as threshold, and the age-grade delay**

|  |  | **ALPHIV living in family settings (n=564)** | | | | |
| --- | --- | --- | --- | --- | --- | --- |
|  |  | **Academic delay (≥ 2 years) or dropout** | |  | **Age-grade delay** | |
|  |  | **ORA (IC95%)** | **p-value**† |  | **ORA (IC95%)** | **p-value**† |
| Sex |  |  |  |  |  |  |
| Female |  | 1 |  |  | 1 |  |
| Male |  | 1.66 [1.03-2.70] | 0.04 |  | 1.99 [1.28-3.10] | 0.002 |
|  |  |  |  |  |  |  |
| Age (years) |  | 1.30 [1.12-1.50] | <0.001 |  | 1.24 [1.08-1.42] | 0.001 |
|  |  |  |  |  |  |  |
| Neurocognitive difficulties |  |  |  |  |  |  |
| No |  | 1 |  |  | 1 |  |
| Yes |  | 2.97 [1.70-5.15] | <0.001 |  | 2.54 [1.50-4.30] | <0.001 |
|  |  |  |  |  |  |  |
| HAZ | |  |  |  |  |  |
| ≥ -2 |  | 1 |  |  | 1 |  |
| < -2 |  | 1.66 [1.03-2.69] | 0.04 |  | 1.81 [1.17-2.81] | 0.01 |
|  | |  |  |  |  |  |
| Ethnic origin |  |  |  |  |  |  |
| Thai |  | 1 |  |  | 1 |  |
| Ethnic minority |  | 5.30 [1.67-16.7] | 0.004 |  | 3.61[1.15-11.17] | 0.03 |
|  |  |  |  |  |  |  |
| Caregiver’s level of education | |  |  |  |  |  |
| Secondary school and above | | 1 |  |  | 1 |  |
| Primary school | | 2.33 [1.14-5.15] | 0.03 |  | 1.71 [0.93-3.28] | 0.09 |
| Never attended school | | 3.24[1.28-8.54] | 0.01 |  | 2.15 [0.94-4.98] | 0.07 |
|  |  |  |  |  |  |  |
| Stigmatization experience(s) at school | | |  |  |  |  |
| No or don’t know | | 1 |  |  | 1 |  |
| Yes once or more | | 2.40 [1.45-398] | <0.001 |  | 1.86 [1.14-3.00] | 0.01 |
|  | | |  |  |  |  |
| ART type |  |  |  |  |  |  |
| NNRTI based |  | 1 |  |  | 1 |  |
| PI based (or other) | | 1.79 [1.07-2.99] | 0.03 |  | 1.88 [1.17-3.02] | 0.01 |
|  |  |  |  |  |  |  |
| Age at ART initiation | |  |  |  |  |  |
| < 9 years |  | 1 |  |  | 1 |  |
| ≥ 9 years |  | 1.02 [0.58-1.79] | 0.95 |  | 1.68 [1.00-2.83] | 0.05 |
|  |  |  |  |  |  |  |
| Type of caregiver | | |  |  |  |  |
| Parent |  | 1 |  |  | 1 |  |
| Grandparent |  | 0.95 [0.52-1.76] | 0.88 |  | 1.01 [0.58-1.78] | 0.96 |
| More distant relative or guardian | | 1.35 [0.74-2.49] | 0.34 |  | 1.40 [0.80-2.46] | 0.23 |
|  |  |  |  |  |  |  |
| Household’s financial situation | |  |  |  |  |  |
| Fair / good / very good |  | 1 |  |  | 1 |  |
| Difficult / very difficult | | 1.10 [0.67-1.77] | 0.71 |  | 0.89 [0.56-1.39] | 0.61 |
|  |  |  |  |  |  |  |
| Public school |  |  |  |  |  |  |
| Yes |  | 1 |  |  | 1 |  |
| No |  | 1.20 [0.48-2.78] | 0.68 |  | 0.90 [0.37-2.02] | 0.80 |
|  |  |  |  |  |  |  |
| Type of living area | |  |  |  |  |  |
| Rural |  | 1 |  |  | 1 |  |
| Urban |  | 1.68 [0.96-2.90] | 0.06 |  | 1.27 [0.75-2.13] | 0.37 |
|  |  |  |  |  |  |  |
| History of hospitalizations | |  |  |  |  |  |
| No |  | 1 |  |  | 1 |  |
| Yes |  | 1.12 [0.69-1.84] | 0.64 |  | 1.35 [0.87-2.14] | 0.19 |

${}^{\dagger}$: Wald test
