# Supplementary material for: School trajectory disruption among adolescents living with perinatal HIV receiving antiretroviral treatments: a case-control study in Thailand
Source: BMC Public Health. 2021 Jan 21;21:189. doi: 10.1186/s12889-021-10189-x (PMC7818931; doi:10.1186/s12889-021-10189-x)
Supplement: Supplementary file 5 — Additional file 5. Factors associated with school trajectory disruption among all adolescents excluding HEU: multivariable analysis. [file 12889_2021_10189_MOESM5_ESM.docx]

|  |  | **Without HEU (n=1372)** | |
| --- | --- | --- | --- |
|  |  |  | |
|  |  | **ORA (IC95%)** | **p-value**† |
| HIV status |  |  |  |
| Controls |  | 1 |  |
| ALPHIV |  | 5.02 [3.62-7.07] | <0.001 |
|  |  |  |  |
| Sex |  |  |  |
| Female |  | 1 |  |
| Male |  | 1.70 [1.27-2.28] | <0.001 |
|  |  |  |  |
| Type of caregiver |  |  |  |
| Parent or grandparent |  | 1 |  |
| More distant relative or guardian |  | 1.50 [1.03-2.16] | 0.03 |
| Institution staff |  | 11.3 [7.90-16.38] | <0.001 |
|  |  |  |  |
| History of hospitalizations |  |  | |
| No |  | 1 |  |
| Yes |  | 1.48 [1.09-2.0] | 0.01 |
|  |  |  |  |
| Age (years) |  | 1.22 [1.13-1.31] | <0.001 |

**Additional file 5. Factors associated with school trajectory disruption among all adolescents excluding HEU: multivariable analysis**

${}^{\dagger}$: Wald test
